# New Strategy for Enhancing Energetic Properties by Regulating Trifuroxan Configuration: 3,4-Bis(3-nitrofuroxan-4-yl)furoxan

**DOI:** 10.1038/s41598-019-39723-z

**Published:** 2019-03-13

**Authors:** Lianjie Zhai, Fuqiang Bi, Yifen Luo, Naixing Wang, Junlin Zhang, Bozhou Wang

**Affiliations:** 1 State Key Laboratory of Fluorine & Nitrogen Chemicals, Xi’an Modern Chemistry Research Institute, Xi’an, 710065 China; 2 Technical Institute of Physics and Chemistry & University of Chinese Academy of Sciences, Chinese Academy of Sciences, Beijing, 100190 China

## Abstract

It is of current development to construct high–performance energetic compounds by aggregation of energetic groups with dense arrangement. In this study, a hydrogen-free high-density energetic 3,4-bis(3-nitrofuroxan-4-yl)furoxan (BNTFO-I) was designed and synthesized in a simple, and straightforward manner. Its isomer, 3,4-bis(4-nitrofuroxan-3-yl)furoxan (BNTFO-IV), was also obtained by isomerization. The structures of BNTFO-I and BNTFO-IV were confirmed by single-crystal X-ray analysis for the first time. Surprisingly, BNTFO-I has a remarkable calculated crystal density of 1983 g cm^−3^ at 296 K, which is distinctly higher than BNTFO-IV (1.936 g cm^−3^, 296 K), and ranks highest among azole-based CNO compounds yet reported. It is noteworthy that BNTFO-I exhibits excellent calculated detonation properties (*v*_D_, 9867 m s^−1^, *P*, 45.0 GPa). The interesting configuration differences of BNTFO-I and BNTFO-IV provide insight into the design of new advanced energetic materials.

## Introduction

Along with the growing demand of military and civilian applications, the design and synthesis new energetic materials with high density and excellent performances have been one of the most exciting and challenging tasks for chemists and materials researchers worldwide^1–5^. Density (*ρ*) is likely the most important parameter to be considered in the design and synthesis of new high density energetic materials (HEDMs)^6^. Performances including detonation velocity and detonation pressure are highly dependent on the density. The detonation velocity is proportional to density and the detonation pressure is proportional to the density squared. Therefore, there is ongoing research and interest in the design and synthesis of HEDMs with higher density. However, the higher density of CHON energetic compounds, the more difficult it is to synthesize it arising from their congested and unstable molecule structure^7–9^. Caged compounds are one of the most dense CHON compounds known due to their polycyclic structures and they became a hot subject-matter at the end of last century^10–12^. Though highly nitrated caged compounds can be very dense, such as hexanitrohexaazaisowurtzitane (CL-20, 2.04 g cm^−3^) and octanitrocubane (ONC,1.98 g cm^−3^)^13^, the synthesis is often expensive and includes multiple steps which make the industrial scale-up and practical use infeasible.

**Figure 1:**
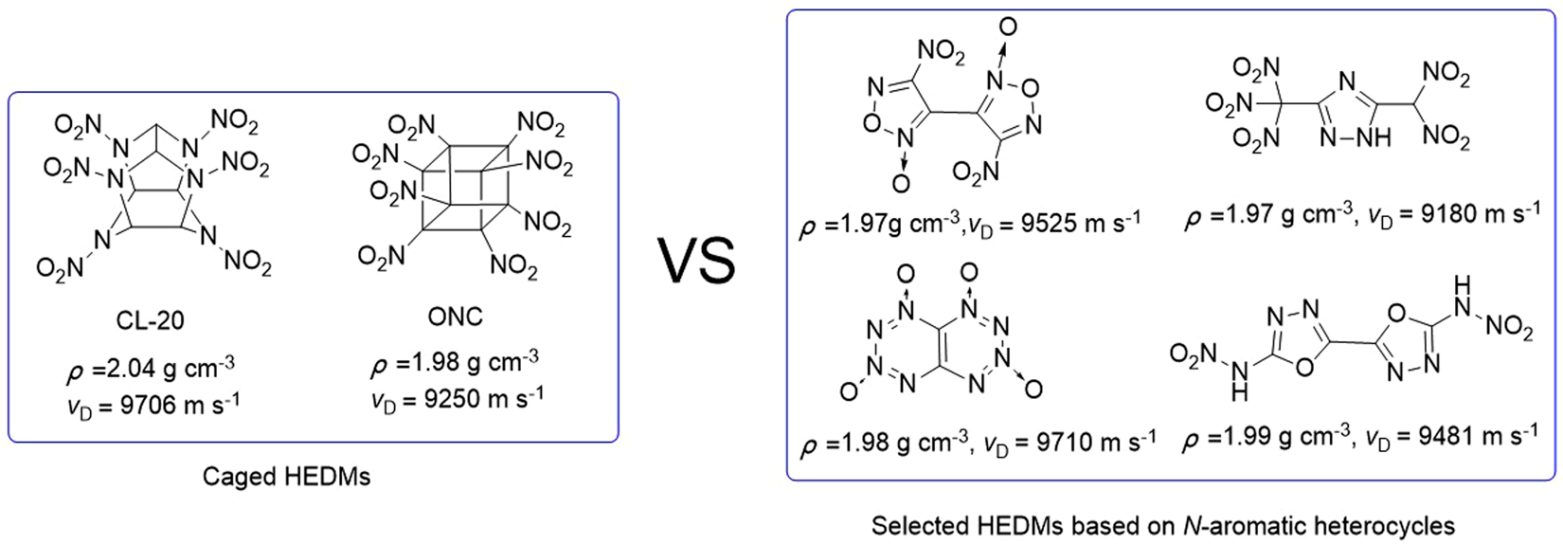
Comparison of properties of caged and azole-based HEDMs.

In recent decades, nitro-substituted nitrogen-containing heterocycles based on imidazole^14,15^, pyrazol^16^, triazole^3,17,18^, tetrazole^19,20^, oxadiazole^21,22^, tirzine and tetrazine derivatives^23–25^ have successfully fulfilled many requirements in the pursuit of HEDMs. In general, not only the nitrogen atom of heterocycle possesses advantages of higher density and better thermal stability over their nonaromatic carbon-substituted analogues, but it also increases the heat of formation due to inherently energetic C–N and N–N bonds contained in the molecule. A variety of novel CHON energetic materials–based heterocycles have been reported as milestone achievements over the past several years, such as 4,4′-dinitro-3,3′-bisfuroxan^26^, 3-trinitromethyl-5-dinitromethyltrizole^27^, tetrazino-tetrazine 1,3,6,8-tetraoxide (TTTO)^28–30^, and 2,2′-dinitramino-5,5′-bi(1,3,4-oxadiazole) (ICM-101)^31^ (Fig. 1).

**Figure 2:**
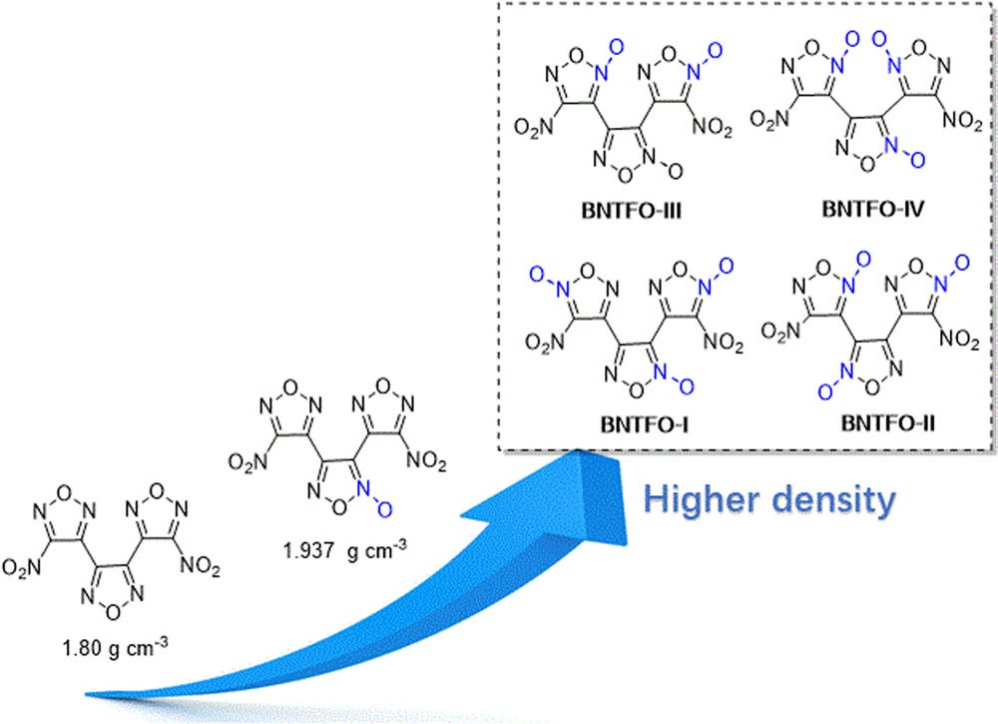
The trends in density with the increase in number of furoxan rings.

Aimed at pushing the limits of density and energy in nitrogen-containing heterocycles, we have been interested in furoxan-based energetic materials that usually have an increased density, oxygen balance and performance of the energetic material in comparison with other five-membered heterocycles^32,33^. Examples of these kinds of molecules have impressively be demonstrated on 3,4-bis(4-nitro-1,2,5-oxadiazol-3-yl)-1,2,5-oxadiazole (LLM-137) and 3,4-bis(4-nitro-1,2,5-oxadiazol-3-yl)-1,2,5-oxadiazole-2-oxide (BNFF, Fig. 2)^34–36^. By replacing a furazan ring with a furoxan ring in LLM-137, the density and detonation velocity of BNFF can be increased by approximately 0.1 g cm^−3^ and 700 ms^−1^, respectively. Thus, the promising properties of BNFF attracted our attention to the dinitro-substituted C-C connected trifuroxan (BNTFO), which are expected to have higher density and superior detonation properties due to the introduction of trifuroxan as a building block.

Interestingly, dinitro-substituted C-C connected trifuroxan has four isomers according to the connection mode of the three furoxans, that is BNTFO-I, BNTFO-II, BNTFO-III and BNTFO-IV (Fig. 2). Recently, Jean’ne M. Shreeve and coworkers have successfully synthesized 3,4-bis(4-nitrofuroxan-3-yl)furoxan (BNTFO-IV)^37^. Inexplicably, BNTFO-IV has a measured density of 1.91 g cm^−3^ (gas pycnometer) even lower than BNTF (1.937 g cm^−3^), which is not in accordance with our assumption. Given BNTFO-IV is the isomer with the lowest density in the four isomers, we believe BNTFO-I has the highest density due to “inverse” molecule configuration compared with BNTFO-IV. Herein, we report the synthesis and characterization of BNTFO-I. Its crystal structure and properties were compared with that of BNTFO-IV. Remarkably, BNTFO-I shows the highest crystal density among nitro-functionalized azole-based CNO compounds reported thus far and superior explosive performance compared to Cl-20.

## Results and Discussion

**Figure 3:**
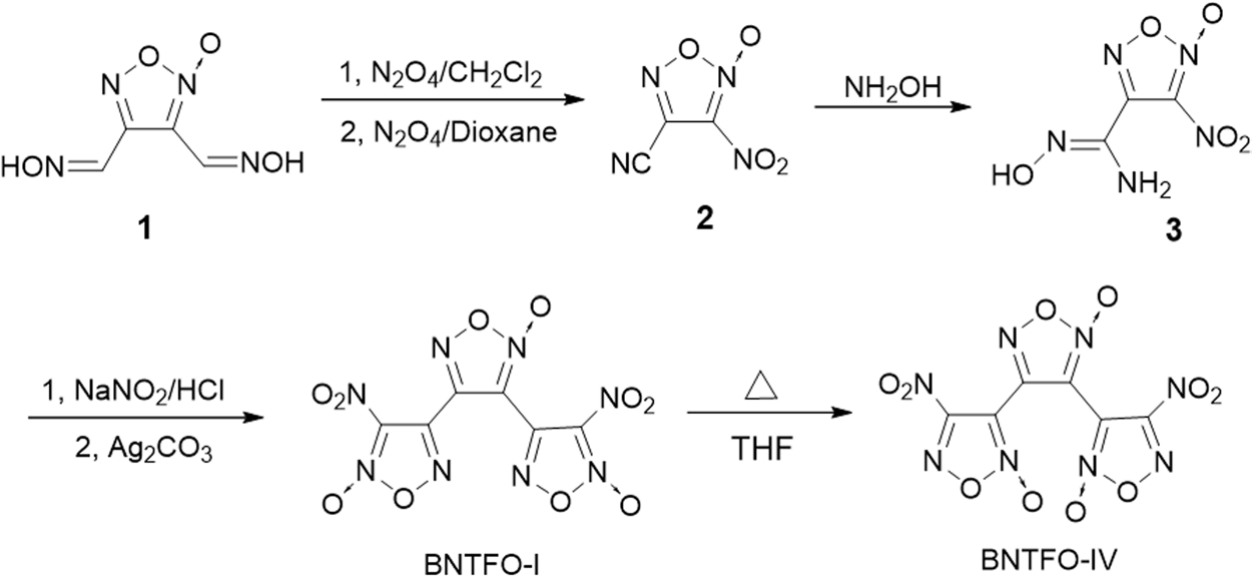
Synthesis of BNTFO-I and BNTFO-IV.

The synthetic pathway to BNTFO-I is shown in Fig. 3. 3,4-Bis(oximomethyl)furoxan (**1**) and 3-nito-4-cyanofuroxan (**2**) were prepared according to a known procedure^38–40^, and **2** was then treated with 50% aqueous hydroxylamine in ethanol to give 3-nitro-4-aminoximidofuoxan (**3**) in moderate yield. Diazotization of **3** with sodium nitrite and concentrated hydrochloric acid gave 3-nitro-4-chloroximidofuoxan. This product does not need to be isolated for the following steps. We originally intended to prepare BNTFO-I by treatment of ether solution of 3-nitro-4-chloroximidofuoxan with aqueous Na_2_CO_3_ or organic weak bases, such as, pyridine and triethylamine, to undergo a dipolar [2 + 3] cycloaddition reaction. However, BNTFO-I was not obtained from the reaction. This might be due to alkaline hydrolysis of nitro group in 3-nitro-4-chloroximidofuoxan induced by relatively strong alkaline Na_2_CO_3_, which led us to investigate the use of slightly weaker base. Ag_2_CO_3_ was selected as the cycloaddition reagent, as it has the attractive property of being both a silver chloride and a weak base in Sn_1_ elimination reactions. Indeed, treatment of 3-nitro-4-chloroximidofuoxan with Ag_2_CO_3_ in THF produced BNTFO-I in high yields of 84% at room temperature overnight. In an effort to obtain crystals suitable for X-ray diffraction of BNTFO-I, the isomer, i.e BNTFO-IV, was formed in some solvents such CH_3_CN, and THF in varying yields.

**Figure 4:**
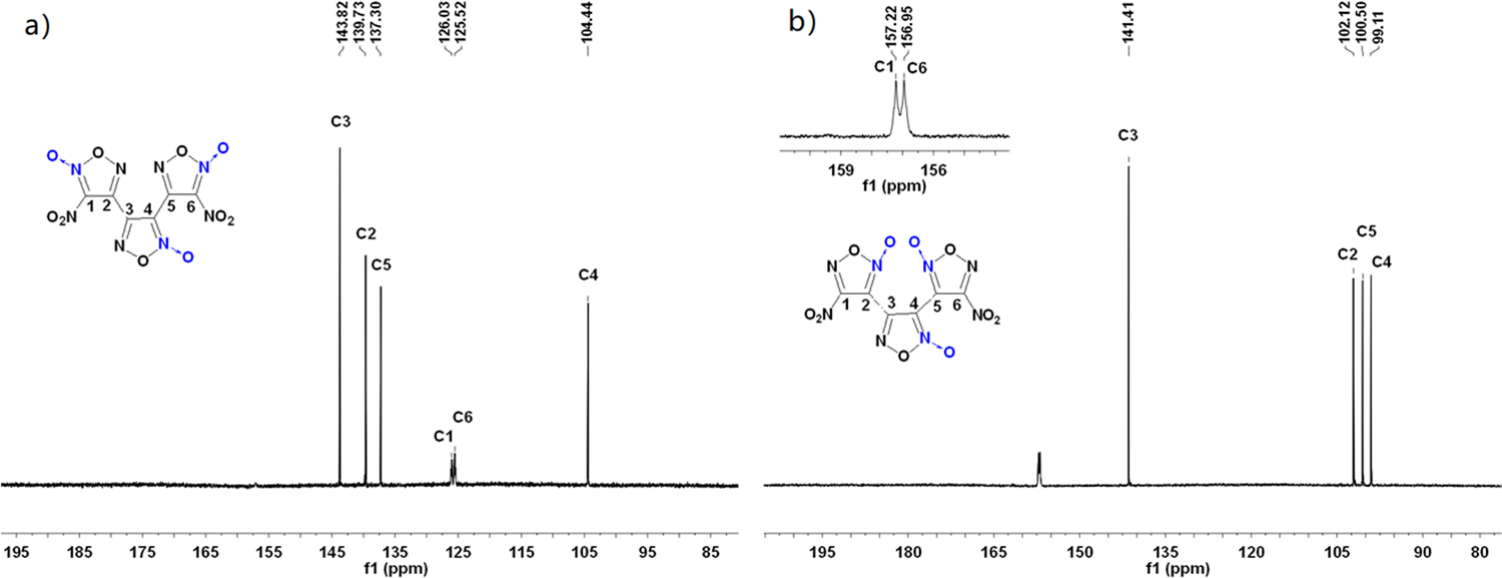
^13^C NMR spectra of BNTFO-I (**a**) and BNTFO-IV (**b**).

The structures of BNTFO-I and BNTFO-IV were confirmed by IR and ^13^C NMR spectroscopy, elemental analysis, and X-ray diffraction. As expected, the ^13^C NMR spectra of BNTFO-I and BNTFO-IV showed six different signals for the chemically different carbon atoms of the molecules, respectively. The carbon atoms in these furoxan rings commonly appear as two resonances; one occurred at adjacent *N*–oxide can be found around 100 ppm, and the other are observed at low-field with a value at around 140 ppm. So the low-field shifted signals appeared at 137~144 ppm belong to C2, C3 and C5 in BNTFO-I, and the high-field resonance signals belong to carbon atoms adjacent *N*–oxide in furoxans (Fig. 4a). Due to the electron withdrawing nature of nitro group, the chemical shifts of C1 and C6 in BNTFO-I were observed at 125.5 and 126.0 ppm, which are clearly shifted downfield compared with that of C4 (*δ* = 104.4 ppm). The same trend was observable for the carbon atoms of BNTFO-IV (Fig. 4b). Three resonances of carbon atoms adjacent *N*–oxide in furoxans (C2, C4 and C5) are observed with a range at 103~99 ppm, while the down-field shifted signals appeared at 157.22 and 156.95 ppm belong to the carbon bonded to NO_2_ and at 141.4 ppm belong to the central furoxan carbon. In addition, every carbon signal was assigned successfully based on GIAO NMR calculation with Gaussian 09^41,42^, which are consistent with our experiment analysis.

**Figure 5:**
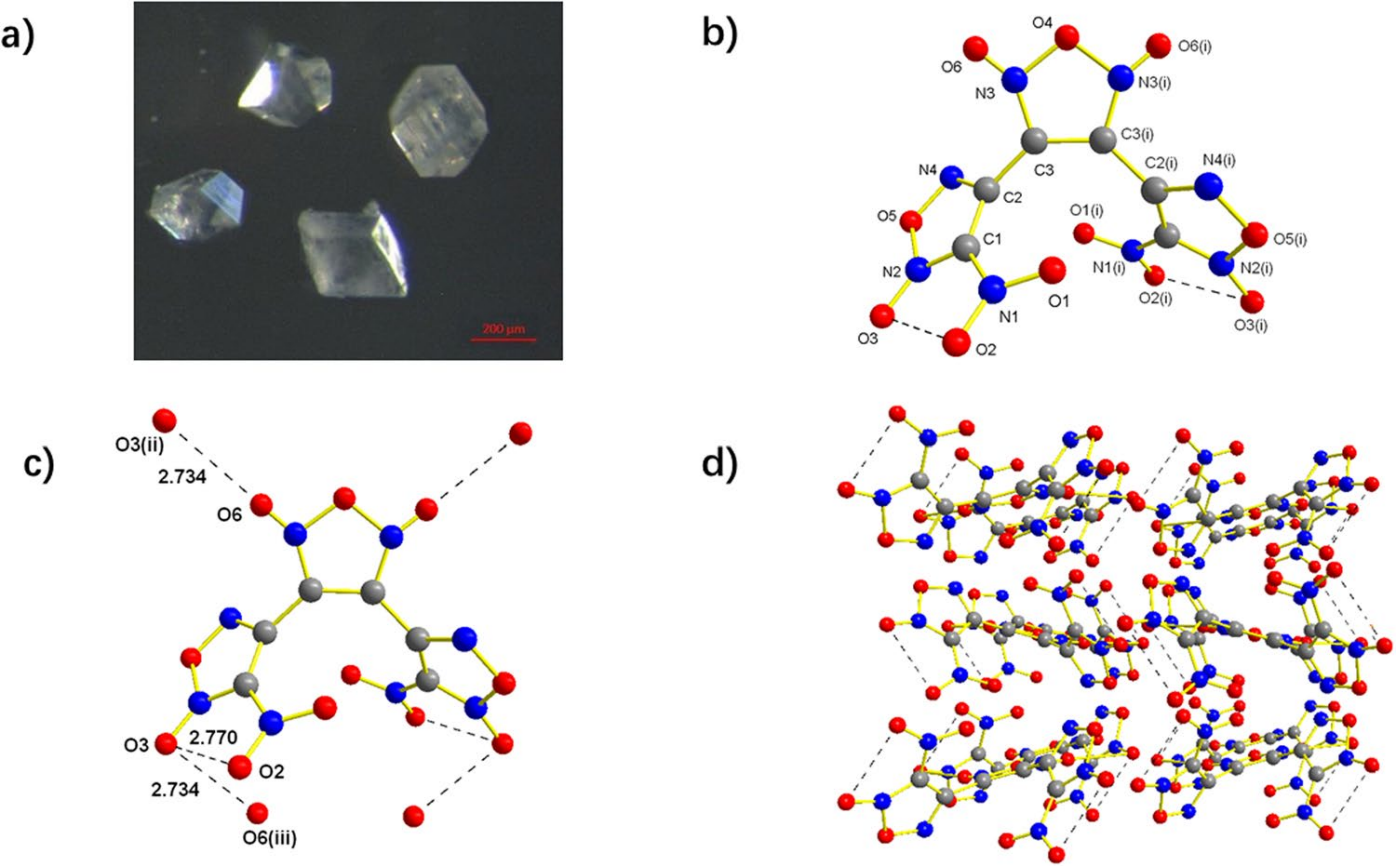
(**a**) Optical microscopic picture of BNTFO-I. (**b**) Crystal structure of BNTFO-I. Symmetry operators: (i) 2 − x, −y, z. c). (**c**) The short contacts around the BNTFO-I molecule. Symmetry operators: (ii) x, y, 1 + z; (iii) x, y, −1 + z. (**d**) Packing diagram of BNTFO-I viewed along the *c* axis.

Crystals of BNTFO-I and BNTFO-IV, suitable for single-crystal X-ray diffraction, were obtained by slow evaporation of CHCl_3_ solutions at room temperature. The crystallographic data and refinement details can be found in the Supporting Information. The morphology of BNTFO-I is depicted in Fig. 5a. The colorless BNTFO-I crystals are beautiful octahedron in shape with a mean dimension of roughly 150 μm × 140 μm × 130 μm. BNTFO-I crystallizes in the orthorhombic space group *Aea*2 with four formula units in the unit cell (Fig. 5b). The central furoxan ring is twisted by 53° towards the adjacent ring plane. The nitro group containing N3 is twisted out of the adjacent ring plane by 14°. Surprisingly, BNTFO-I show a remarkable high density of 1.983 g cm^−3^ at 296 K. Among the nitro-functionalized azole-based CNO compounds, BNTFO-I displays the highest X–ray density at ambient temperature to the best of our knowledge. The remarkably high densities can be rationalized in terms of considerable weak attractive electrostatic interactions. As can be seen from Fig. 5c, each BNTFO-I molecule is surrounded by other six molecules via intermolecular short O···O contacts. The distances O3···O6iii are about 2.73 nm, which are clearly shorter than the sum of van der Waals radii (*r*_w_ (O) + *r*_w_ (O) = 3.20 Å)^43^. Additional, the distance O2···O3 is about 2.77 nm within the molecules of BNTFO-I, indicating a strong intramolecular contacts between O2 of the nitro groups and O3 of furoxan rings. The dense 3D network is formed further by the interactions (Fig. 5d).

**Figure 6:**
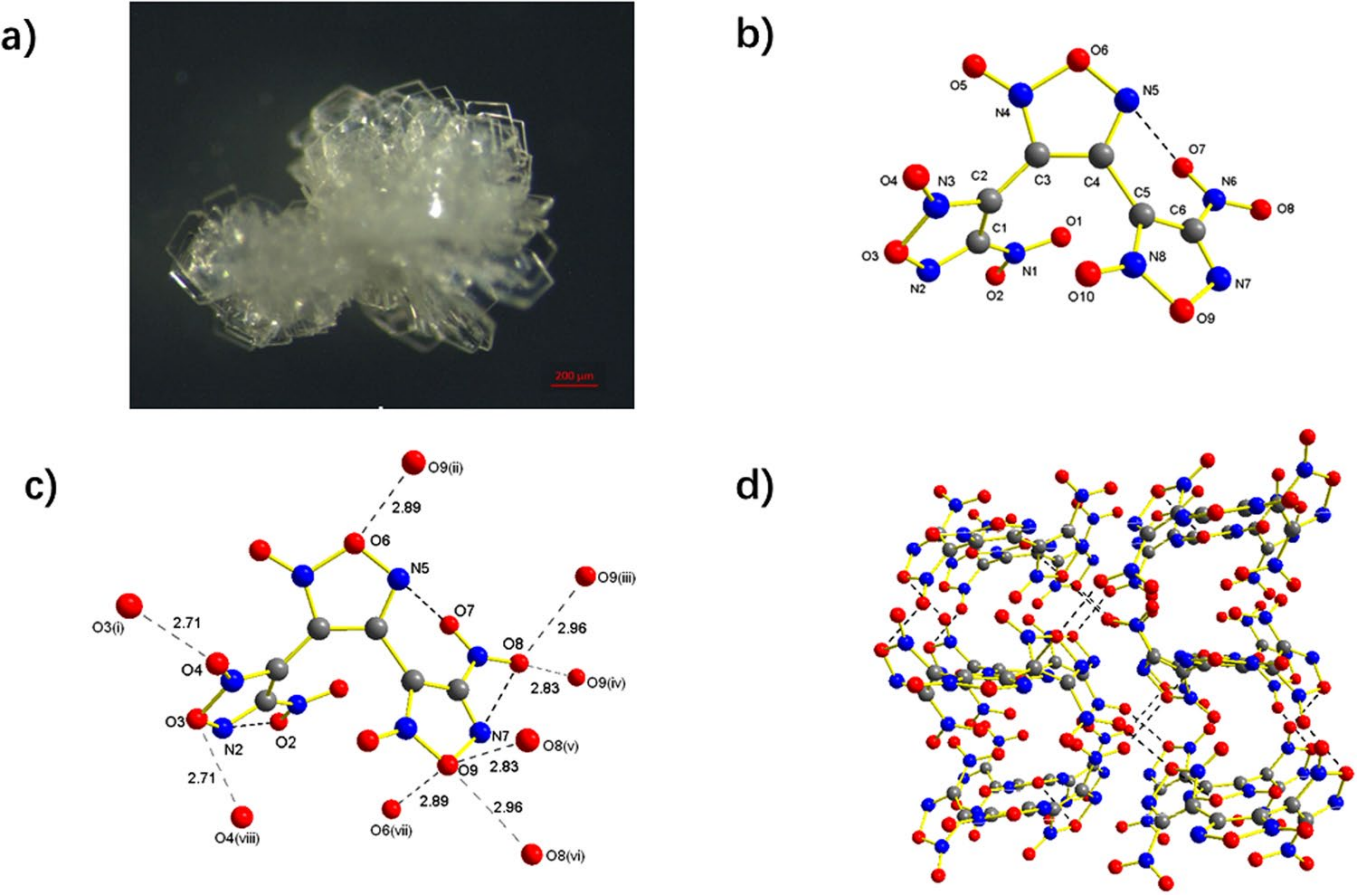
(**a**) Optical microscopic picture of BNTFO-IV. (**b**) Crystal structure of BNTFO-IV. (**c**) The intermolecular short contacts around the BNTFO-IV molecule. Symmetry operators: (i) 2.5 − x, y, 0.5 + z; (ii) x, y, 1 + z; (iii) 2 − x, 1 − y, 0.5 + z; (vi) −0.5 + x, 1 − y, z; (v) 0.5 + x, 1 − y, z; (vi) 2 − x, 1 − y, −0.5 + z; (vii) x, y, −1 − z; (viii) 2.5 − x, 1 − y, −0.5 + z. (**d**) Packing diagram of BNTFO- IV viewed along the *c* axis.

The BNTFO-IV crystals are rectangle in shape and packed into a flower-like structure, Fig. 6a. BNTFO-IV crystallizes in the orthorhombic space group *Pca*2(1) with four formula units in the unit cell. The calculated density at 296 K is 1.936 g cm^−3^, which is notably lower than that of BNTFO-I. Similarly, a series of intramolecular as well as intermolecular short contacts were observed in the crystal. The intramolecular short contacts occur between the oxygen atoms (O7) of the nitro groups and nitrogen atoms of the furoxan ring (N5) (Fig. 6b), while the intermolecular short contacts are only observed between the oxygen atoms (Fig. 6c). The central furoxan ring is twisted by 45.5° and 58.3°, respectively, towards the adjacent ring planes. The nitro group containing N3 is twisted out of the adjacent ring plane by 17.2°, and the other nitro group is twisted by 14.8°. The relatively low crystal density of BNTFO-IV compared to BNTFO-I can be explained by lager dihedral angles between furoxan rings and nitro groups, as well as relatively loose 3D network (Fig. 6d).

The physical and energetic properties of BNTFO-I are summarized in Table 1. The thermal behaviour is determined by DSC at a heating rate of 5 °C min^−1^. BNTFO-I exhibits a melting point at 129.6 °C and a decomposition temperature of 130.7 °C which is slightly lower than BNTFO-IV. The sensitivities towards impact and friction were determined experimentally according to BAM method. The impact and friction sensitivities of BNTFO-I are 3 J and 35 N, similar with that of BNTFO-IV. BNTFO-I have higher positive heats of formation (Δ_f_*H* = 667.8 kJ mol^−1^) than BNTFO-IV, although they all possess three furoxan rings. BNTFO-I has a good oxygen balances of −9.3% that is similar to that of CL-20 (−10.9%), indicating that the chemical energy stored in the compound can fully be utilized. The detonation properties of BNTFO-I were evaluated by using the EXPLO5 6.04 program. BNTFO-I shows remarkable values (*v*_D_ = 9867 m s^−1^, *P* = 45 GPa) that are superior to those of BNTFO-IV (*v*_D_ = 9503 m s^−1^, *P* = 40.8 GPa) and comparable to those of TKX-50 (*v*_D_ = 9698 m s^−1^, *P* = 42.4 Pa) and Cl-20 (*v*_D_ = 9706 m s^−1^, *P* = 45.2 Pa). Noteworthy, these performances make it promising as one of highest azole-based CNO materials.

**Table 1:** Physical and energetic properties of BNTFO-I compared with BNTFO-IV, TKX-50, and CL-20.

Compd	BNTFO-I	BNTFO-IV^37^	TKX-50^49^	CL-20^37^
Formula	C_6_N_8_O_10_	C_6_N_8_O_10_	C_2_H_8_N_10_O_4_	C_6_H_6_N_12_O_12_
*M*/g mol^−1^	344	344	236	438
IS/J^a^	3	3	20	4
FS/N^b^	35	40	120	48
N + O/%^c^	79	79	86	82
*Ω*(CO_2_)/%^d^	−9.3	−9.3	−27	−10.9
*T*_dec_/°C^e^	130.7	146.5	221	195
*ρ/*g cm^−3f^	1.98	1.91	1.88	2.04
Δ_f_*H*/kJ mol^−1g^	667.8	579.2	446.6	403.2
*P/*Gpa^h^	45.0	40.8	42.4	45.2
*v*_*D*_*/*m s^−1i^	9867	9503	9698	9706

^a^Impact sensitivity. ^b^Friction sensitivity. ^c^Nitrogen and oxygen content. ^d^Oxygen balance assuming the formation of CO_2_. ^e^Thermal decomposition temperature (onset, DSC, 5 °C min^−1^). ^f^Gas pycnometer (25 °C). ^g^Calculated molar enthalpy of formation. ^h^Detonation pressure. ^i^Detonation velocity.

**Figure 7:**
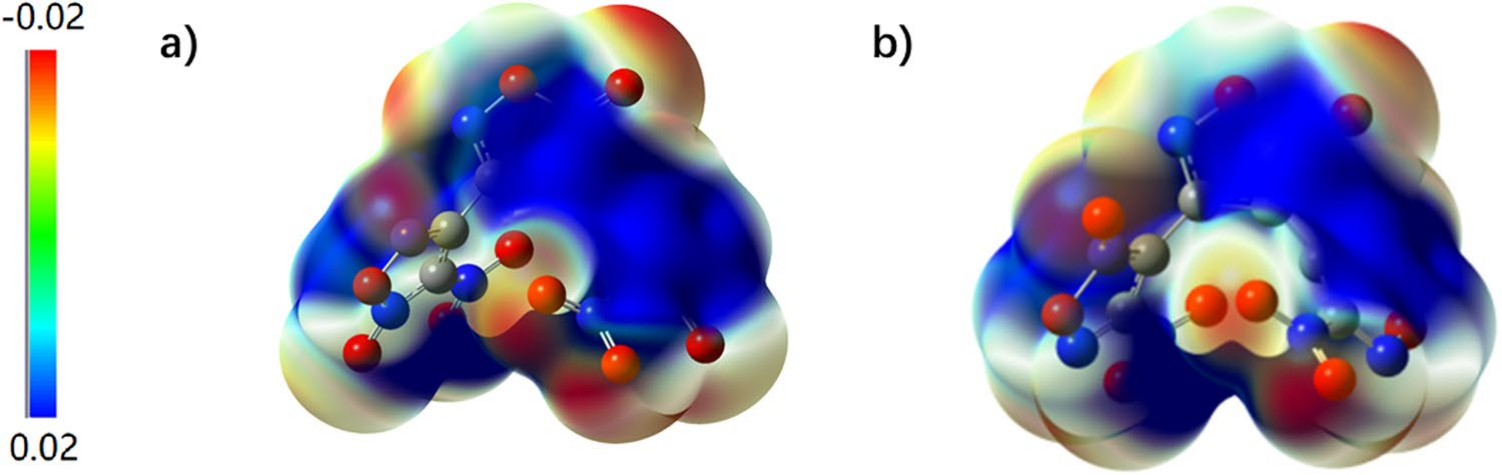
Calculated [B3LYP/6–31 + G(d,p)] electrostatic potential of the compound BNTFO-I and BNTFO-IV isosurface of electron density is shown between −0.02 hartree (electron-rich regions) and +0.02 hartree (electron-poor regions).

In order to obtain a better understanding of the relationship between structure and sensitivities, electrostatic potential (ESP) of compounds BNTFO-I and BNTFO-IV were calculated on the B3LYP/6–31 + g(d,p) level of theory using the optimized gas-phase structure with the Gaussian09 software^41^. The computed ESP which has recently been reported and extensively used is generally related to the impact sensitivity of the bulk energetic material^44,45^. According to Murray and Politzer^46,47^, the charge distribution of the molecular surface of impact-sensitive compounds corresponds to electron-poor areas above covalent bonds. The surface extent and the intensity of these electropositive potential surfaces are coherent with higher impact sensitivities. Figure 7 shows the ESP for the 0.001 electron/bohr^3^ isosurface of the electron density. As can be seen in Fig. 7, large and strong positive potentials, as well as high charge separation are observed which are in good accord with the experimental results of relatively high impact sensitivity and friction sensitivity of BNTFO-1 and BNTFO-IV. Especially it can be clearly seen that a higher charge separation and larger positive region can be found for compound BNTFO-I than that of BNTFO-IV. In addition, the total energies of BNTFO-I, BNTFO-II, BNTFO-III and BNTFO-IV were calculated at the MP2/6–311 + + G** level, and the values are −1415.1813458, −14151942923, −14151915227, and −1415.1979171 a.u., respectively.

## Conclusions

In summary, a new CNO compound BNTFO-I has been synthesized and converted to BNTOF-IV by isomerization in a simple and straightforward approach. The single–crystal X–ray structures of BNTOF-I and BNTOF-IV were investigated and compared. Amazingly, BNTFO-I has the super–high crystal density (1.983 g·cm^−3^ at 296 K) relative to other heteroaromatic-based CNO compounds. Its remarkable high densities can be rationalized by various intra- and intermolecular interactions. The sensitivities and thermal behavior of BNTOF-I are similar to BNTFO-IV. In addition, BNTOF-I has outstanding energetic properties, which are comparable to CL–20, much better than those of RDX and HMX, suggesting that this compound has a high potential application in solid rocket propellants and explosives. The interesting molecular structures based on trifuroxan as the building blocks provide a new strategy for construction of a new family of HEDMs.

## Methods

### General information

Unless otherwise specified, all commercially available reagents were purchased from chemical suppliers without further purification. ^1^H and ^13^C NMR spectra were recorded on 500 MHz (Bruker AVANCE 500) nuclear magnetic resonance spectrometers. The melting and decomposition points (onset) were obtained on a differential scanning calorimeter (TA Instruments Company, Model DSC-Q200) at a flow rate of 50 mL min^−1^. About 0.5 mg of the sample was sealed in aluminum pans for DSC. Infrared spectra were obtained from KBr pellets on a Nicolet NEXUS870 Infrared spectrometer in the range of 4000–400 cm^−1^. Elemental analyses (C, H and N) were performed on a VARI-El-3 elementary analysis instrument. The morphology was examined by field emission scanning electron microscopy (FE-SEM, Ultra-55, Carl Zeiss, Germany). High-resolution mass spectra (HRMS) were obtained by EI. Column chromatography was performed on silica gel (200–300 mesh).

### X-ray crystallography

The single crystal X-ray experiment was performed on a Bruker Apex II CCD diffractometer equipped with graphite-monochromatized Mo K*α* radiation (*λ* = 0.71073 Å) using *ω* and *φ* scan mode. The structures were solved by the direct method using SHELXTL and refined by means of full-matrix least-squares procedures on *F*^2^ with the programs SHELXL-97^48^. All non-hydrogen atoms were refined with anisotropic displacement parameters. Experimental details of crystal data, data collection parameters and refinement statistics are summarized in Table S1. The crystal structure has been deposited in the Cambridge Crystallographic Data Centre with CCDC 1864221 for BNTFO-I and 1864222 for BNTFO-IV. These data can be obtained free of charge from The Cambridge Crystallographic Data Centre via www.ccdc.cam.ac.uk/data_request/cif.

### Synthesis of 1 and 2

Compounds **1** and **2** prepared were prepared according to the literature procedure^38–40^.

### Synthesis of 3-Nitro-4-aminoximidofuroxan (3)

3-Nitro-4-cyanofuroxan (3.1 g, 20 mmol) was dissolved in 80 mL of methanol, to which 1.3 g (20 mmol) of 50% hydroxylamine solution was added dropwise within 30 min at −15 °C. After the solvent was removed under reduced pressure, the resident was dissolved in 30 mL of diethyl ether and washed with 100 mL of ice water. The ether solution was dried over magnesium sulfate and the solvent was removed under reduced pressure to obtain **2** (2.3 g, 62%) as yellow oily liquid.

### Synthesis of 3,4-Bis(3-nitrofuroxan-4-yl)furoxan (BNTFO-I)

3-Nitro-4-aminoximidofuroxan (1.9 g, 10 mmol) was dissolved in the mixtures of concentrated hydrochloric acid (9 mL) and water (16 mL) at room temperature. Sodium nitrite (0.8 g, 11 mmol) in water (4 mL) was added dropwise to the stirred solution of **2** at 0 °C. After stirring for 3 h, diethyl ether (60 mL) was added to the mixture. Then the diethyl ether solution was separated and removed under reduced pressure, the residue was dissolved in 20 mL of THF. Ag_2_CO_3_ (1 g, 3.6 mmol) was added to the THF reaction mixture at 0 to 5 °C while stirring for 2 hours and then the insoluble solid by filtration. The filtrate was concentrated and the residue was subjected to column chromatography on silica gel using ethyl acetate–pentane (v/v = 1: 10) as eluent to isolate the colorless crystals BNTFO-I (1.13 g, 67%).

### Synthesis of 3,4-Bis(4-nitrofuroxan-3-yl)furoxan (BNTFO-IV)

BNTFO-I (0.5 g) was dissolved in THF (20 mL). After the solution was heated at reflux for 3 h, the solvent was removed and the residue was recrystallization from CHCl_3_ to give BNTFO-IV as colorless solid (0.21 g, 42%).

### 3-Nitro-4-aminoximidofuroxan (3)

yellow oily liquid, 62% yield (2.3 g), R_f_ = 0.38 (petroleum ether/ethyl acetate = 4/1). IR (KBr): $\tilde{v}$ *v* = 3489, 3385, 1733, 1647, 1590, 1558, 1536, 1449, 1368, 1321, 1262, 1106, 1020, 855, 821, 753 cm^−1^. ^1^H NMR (CD_3_CN-*d*^6^): *δ* = 8.70(s, 1 H, -OH), 5.45(s, 2 H, -NH_2_) ppm. ^13^C NMR (CD_3_CN-*d*^6^): *δ* = 144.62, 138.10, 124.85. Elemental analysis calcd (%) for C_3_H_3_N_5_O_5_: C 19.06, H 1.60, N 37.04. Found: C 19.15, H 1.71, N 36.84.

### 3,4-Bis(3-nitrofuroxan-4-yl)furoxan (BNTFO-I)

colorless crystals, 67% yield(1.13 g), R_f_ = 0.42 (petroleum ether/ethyl acetate = 10/1). *T*_melt_ = 127.6 °C, *T*_dec (onset)_ = 130.7 °C. IR (KBr): $\tilde{v}$  *v* = 1657, 1616, 1557, 1527, 1477, 1447, 1381, 1356, 1335, 1275, 1133, 1085, 1030, 984, 950, 860, 831, 792, 761, 744, 712, 692, 646, 592, 511, 477 cm^−1^. ^13^C NMR (DMSO-*d*^6^): *δ* = 143.72, 139.65, 137.27, 126.03, 125.52, 104.44. Elemental analysis calcd (%) for C_6_N_8_O_10_: C 20.94, N 32.56. Found: C 21.09, N 32.33. HRMS (EI, m/z) calcd. for C_6_N8O_10_ [M]^+^ 343.9737, found 343.9738.

### 3,4-Bis(4-nitrofuroxan-3-yl)furoxan (BNTFO-IV)

colorless crystals, 42% yield (0.21 g), R_f_ = 0.40 (petroleum ether/ethyl acetate = 10/1). *T*_melt_ = 145.4 °C, *T*_dec (onset)_ = 146.5 °C. IR (KBr): $\tilde{v}$ *v* = 1661, 1636, 1617, 1566, 1544, 1488, 1453, 1425, 1414, 1335, 1302, 1244, 1202, 1102, 1081, 1052, 983, 950, 834, 797, 767, 753, 740, 701, 673, 637, 537, 476, 455 cm^−1^. ^13^C NMR (DMSO-*d*^6^): *δ* = 157.22, 156.95, 141.41, 102.12, 100.05, 99.11. Elemental analysis calcd (%) for C_6_N_8_O_10_: C 20.94, N 32.56. Found: C 21.05, N 32.41. HRMS (EI, m/z) calcd. for C_6_N8O_10_ [M]^+^ 343.9737, found 343.9741.

## Supplementary information

Supporting info

## Data Availability

The data that support the findings of this study are available from the corresponding authors on request.
