# Supplementary material for: New Strategy for Enhancing Energetic Properties by Regulating Trifuroxan Configuration: 3,4-Bis(3-nitrofuroxan-4-yl)furoxan
Source: Sci Rep. 2019 Mar 13;9:4321. doi: 10.1038/s41598-019-39723-z (PMC6416340; doi:10.1038/s41598-019-39723-z)
Supplement: Supplementary file 1 — Supporting info [file 41598_2019_39723_MOESM1_ESM.pdf]

New Strategy for Enhancing Energetic Properties by Regulating Trifuroxan Configuration: 3,4-Bis(3-nitrofuroxan-4-yl)furoxan

Lianjie Zhai[1], Fuqiang Bi[1], Yifen Luo[1], Naixing Wang[2,*], Junlin Zhang[1], and Bozhou Wang [1,*]

[1]State Key Laboratory of Fluorine & Nitrogen Chemicals, Xi'an Modern Chemistry Research Institute, Xi'an, 710065, China;
[2]Technical Institute of Physics and Chemistry & University of Chinese Academy of Sciences, Chinese Academy of Sciences, Beijing, 100190, China.

Correspondence and requests for materials should be addressed to B. W.(wbz600@163.com) or N. W. (nxwang@mail.ipc.ac.cn)

1. Crystallographic data

Table S1. Crystallographic data for compounds BNTFO-I and BNTFO-IV.

Compounds	**BNTFO-I**	**BNTFO-IV**
Empirical formula	$C_6N_8O_{10}$	$C_6N_8O_{10}$
Formula weight	344.14	344.14
Crystal system	Orthorhombic	Orthorhombic
space group	Aea2	Pca2(1)
a (Å)	9.478(3)	9.9664(10)
b (Å)	16.913(6)	16.5839(17)
c (Å)	7.191(3)	7.1419(8)
α (°)	90	90
β (°)	90	90
γ (°)	90	90
V (Å^3)	1152.7(7)	1180.4(2)
Z	4	4
D_c (g·cm^{-3})	1.983	1.936
T (K)	296(2)	296(2)
μ (mm^{-1})	0.191	0.187
F(000)	688	688
Reflections collected/unique	2930/1152	11489/2709
R(int)	0.0414	0.0683
GOF on F^2	1.058	1.051
R_1 [$I > 2\sigma(I)$]	0.0631	0.0783
wR_2 ($I > 2\sigma(I)$)	0.1434	0.1998
CCDC	1864221	1864222

2. Copies of NMR Spectra

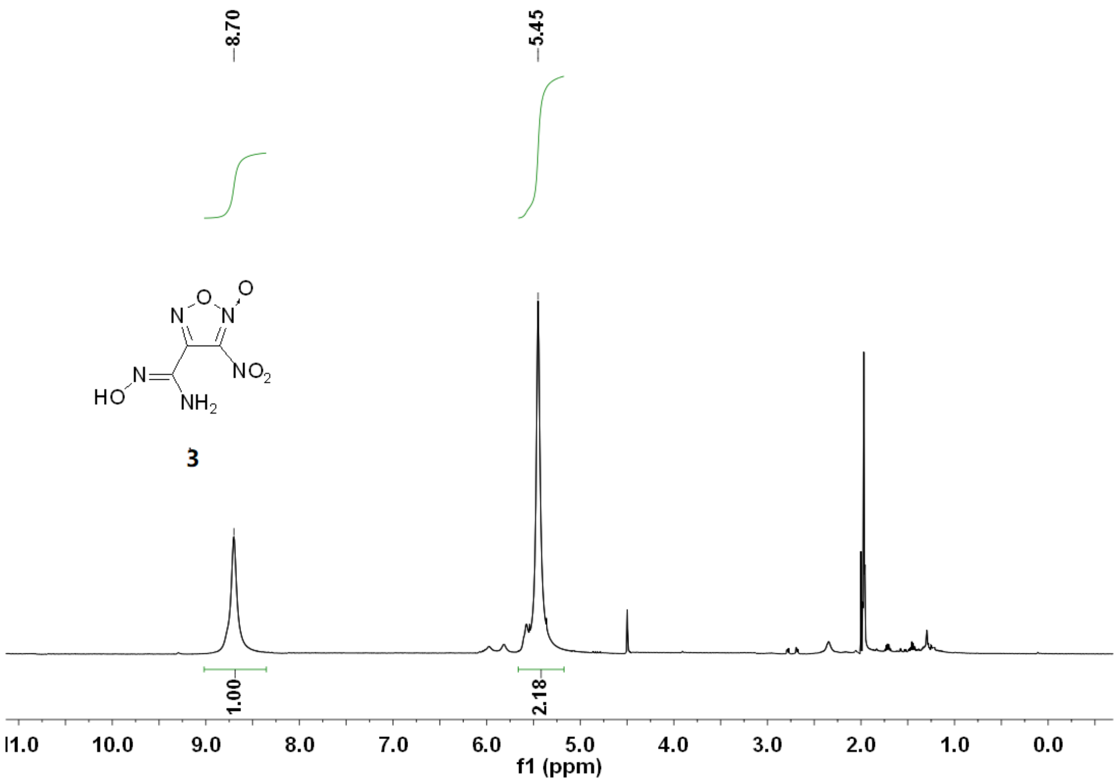

Figure S1. ^{1}H NMR spectrum of **3**

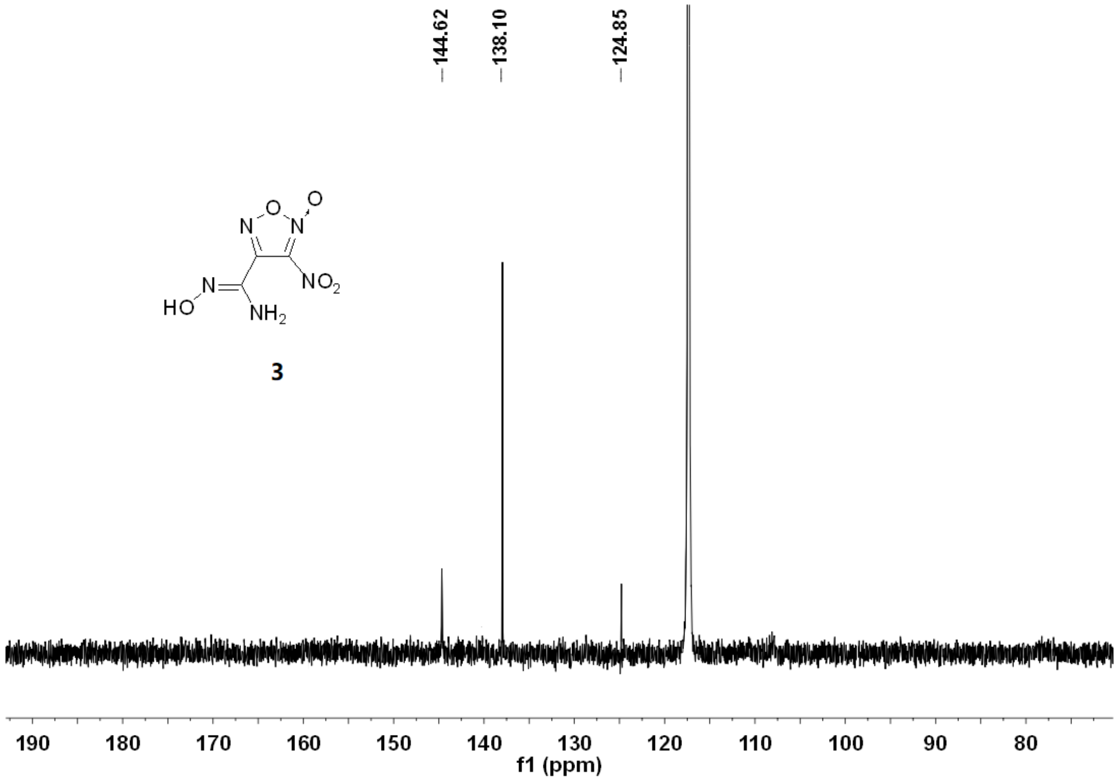

Figure S2. ^{13}C NMR spectrum of **3**

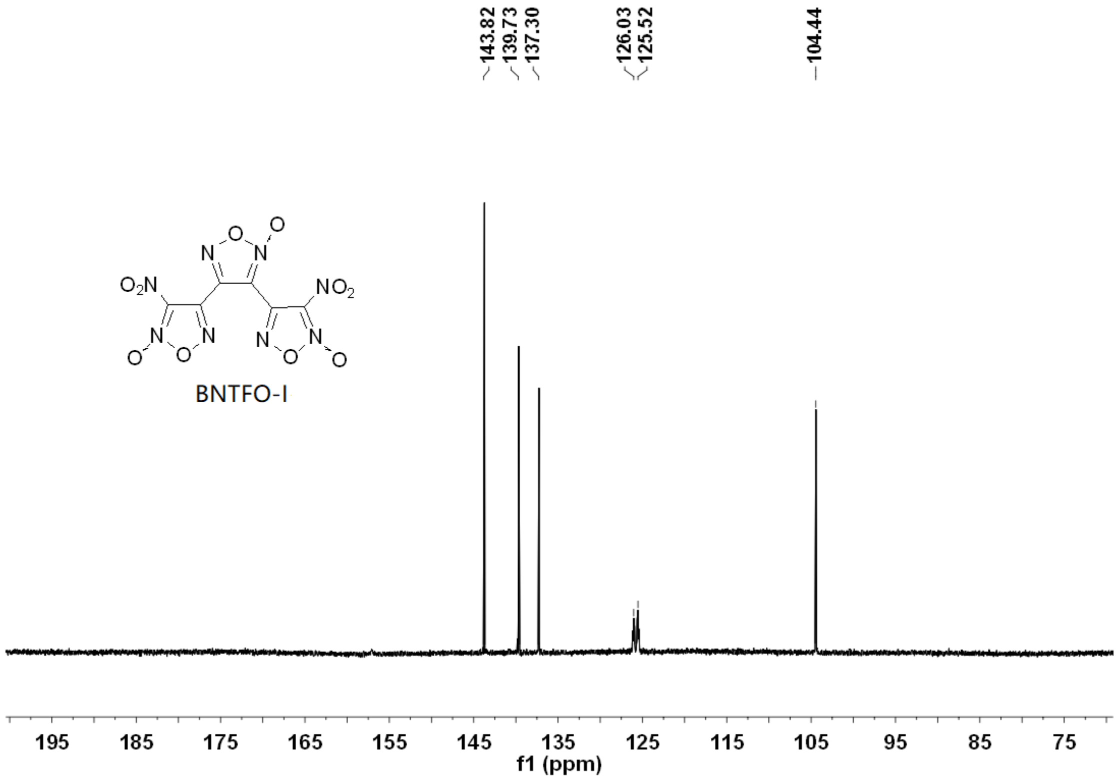

Figure S3. ^{13}C NMR spectrum of BNTFO-I

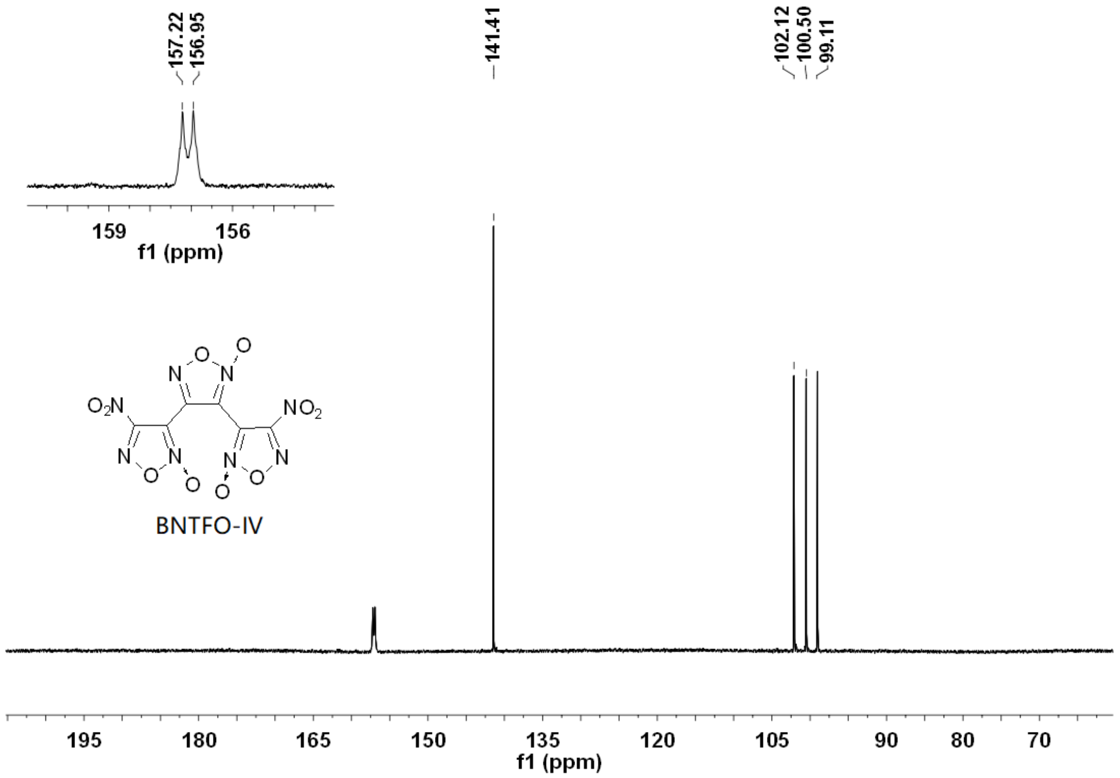

Figure S4. ^{13}C NMR spectrum of BNTFO-IV

3. Copies of DSC Curves

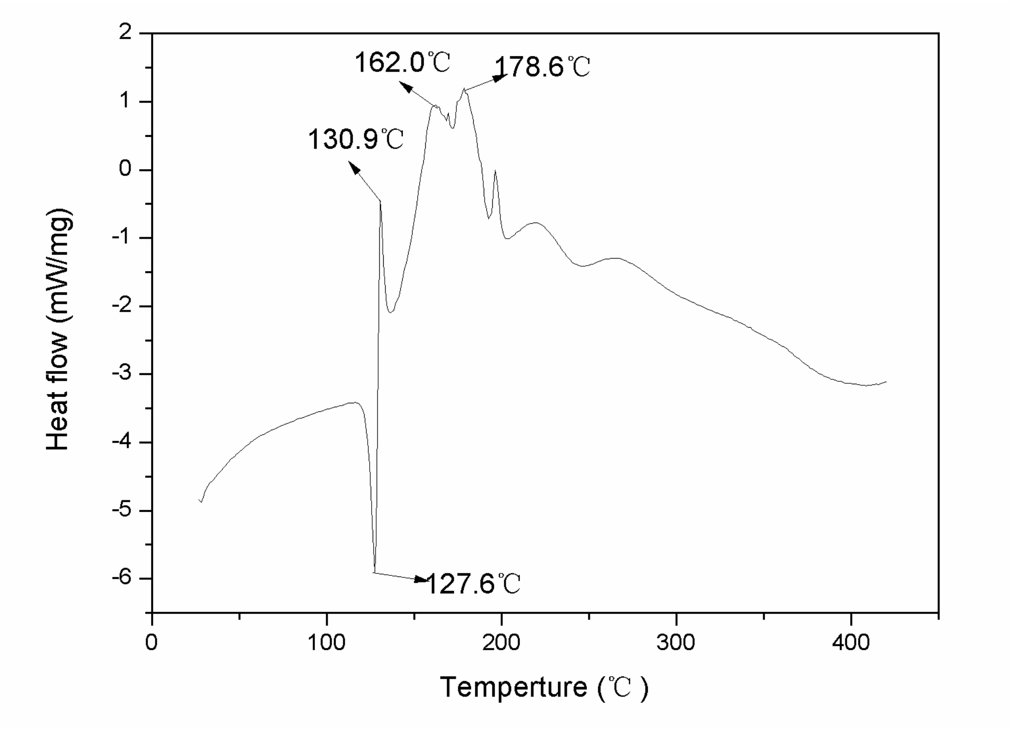

Figure S5. DSC curve of compound BNTFO-I at the heating rate of 5 °C·min^{-1}.

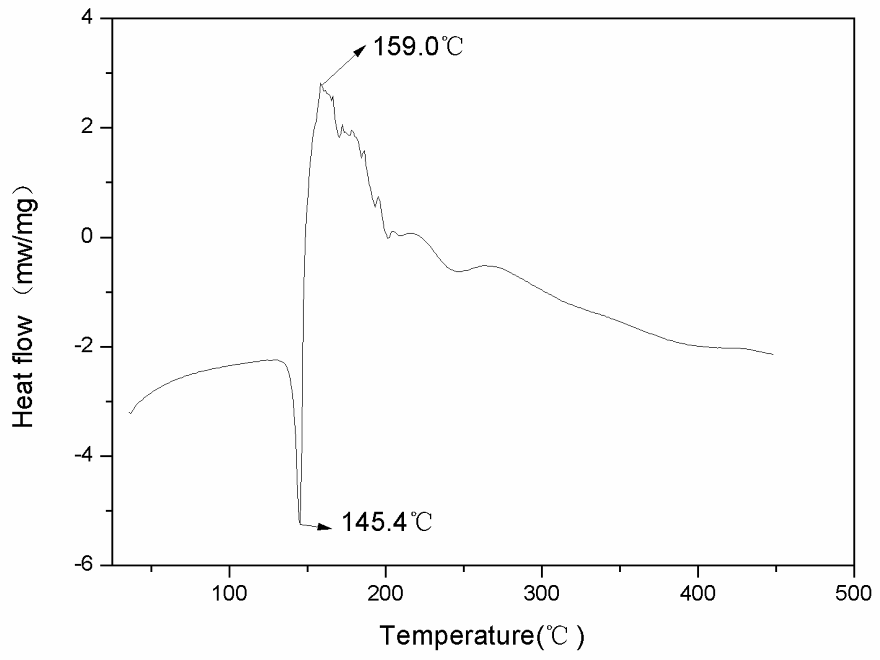

Figure S6. DSC curve of compound BNTFO-IV at the heating rate of 5 °C·min^{-1}.

4. Copies of HRMS Spectra

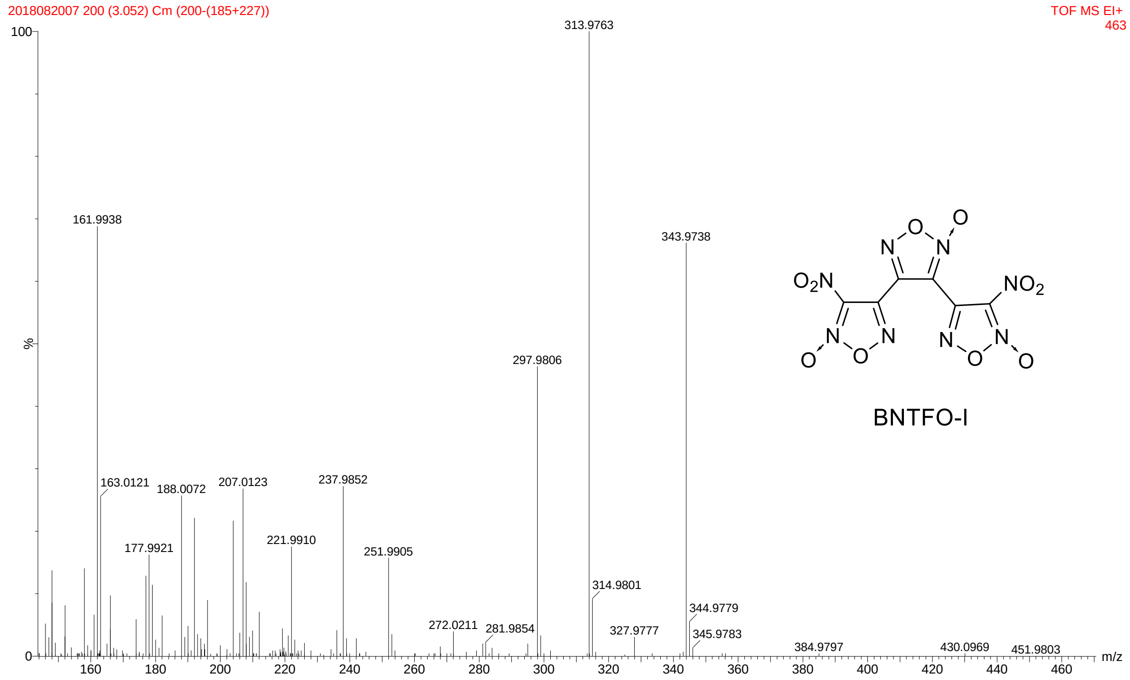

Figure S7. HRMS spectrum of BNTFO-I

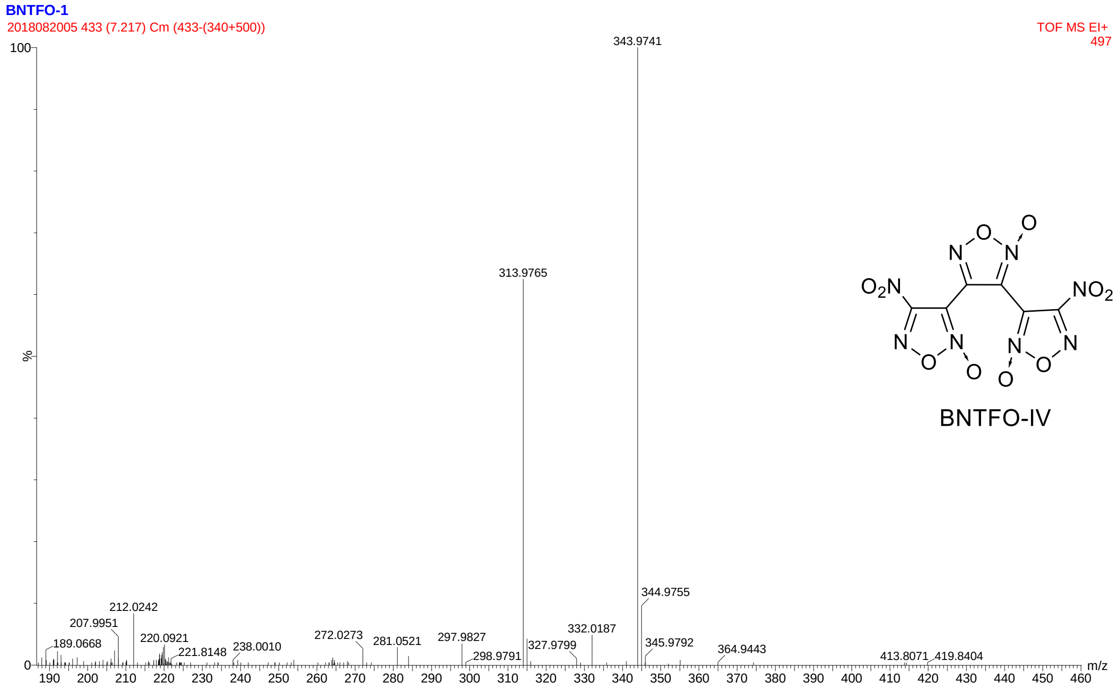

Figure S8. HRMS spectrum of BNTFO-IV

5. DFT Calculations

All quantum chemical calculations were carried out using the Gaussian 09 (Revision A.02) program package[1] and visualized by GaussView 5.08.[2] The geometric optimization and frequency analyses of the structures were carried out using the B3LYP functional with 6–31+G** basis set,[3] and single energy points were calculated at the MP2/6–311++G** level.[4] All of the optimized structures were characterized to be true local energy minima on the potential energy surface without imaginary frequencies.

The heat of formation was determined using an isodesmic reaction (Figure S9). The heats of formation of other compounds in Scheme S1 were determined from the NIST WebBook.[5] Some parameters (E, *ZPE*, H, HOF) were listed in Table S2.

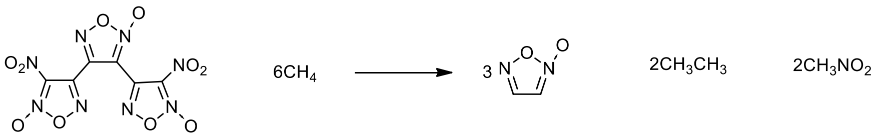

BNTFO-I

Figure S9. Isodesmic reactions for calculating heats of formation for BNTFO-I.

Table S2. Calculated total energy, zero-point energy, values of the correction and heats of formation.

Compound	ZPE[a]	H_{cor}[b]	E_{B3LYP}[c]	E_{MP2}[d]	H[e]	HOF ($kJ\cdot mol^{-1}$)
CH_4	0.044793	0.048605	-40.5261442	-40.3796224	-40.33280912	-74.9[f]
CH_3CH_3	0.074599	0.079027	-79.8416413	-79.5716299	-79.49558686	-84.0[f]
CH_3NO_2	0.049856	0.055129	245.0287319	-244.473453	-244.4203182	-81.0[f]
Furoxan	0.049510	0.054821	-337.2249822	-336.4521556	-336.399315	225.3[g]
BNTFO-I	0.113243	0.133109	-1418.2293111	-1415.1813458	-1415.052767	743.0

[a] Zero-point correction; [b] Thermal correction to Enthalpy; [c] Total energy (B3LYP); [d] Total energy (MP2); [e] $H=E_{MP2}+H_{cor}-(1-0.96)ZPE$; [f] Data from NIST; [g] Calculated by atomization at the CBS-4M level.[6,7]

The solid state enthalpy of formation for neutral compounds can be estimated by subtracting the heats of sublimation from gas phase heats of formation. On the basis of the literatures [8], the heat of sublimation can be estimated with Trouton's rule according to eq 1, where T represents either the melting point or the decomposition temperature when no melting occurs prior to decomposition:

$\boldsymbol{\Delta} H_{sub}=188/J\ mol^{-1}\ K^{-1}\times T$

(1)

Cartesian Coordinates of Optimized geometries

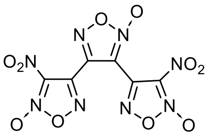

BNTFO-I

O	-3.18822000	-1.01637900	1.21188500
O	-0.46585000	3.61675900	-0.09610700
O	3.52828800	0.18270300	-1.21646700
O	-2.65629000	2.98669900	0.17503300
N	-2.54692900	0.18042200	1.24853500
N	-2.66821500	-1.80079300	0.09501800
N	-1.50738000	2.61192800	0.07371000
N	0.72662400	2.98723500	-0.20394900
N	2.49837600	1.06989200	-1.28727800
N	3.32168600	-0.69513800	-0.07017500
C	-1.73967800	-1.01146700	-0.47152600
C	-1.67895700	0.21219200	0.26775500
C	-0.88587900	1.42014300	0.05102800
C	0.50747700	1.69625600	-0.12604700
C	1.67611100	0.81816000	-0.30041900
C	2.16643000	-0.27555200	0.47573600
O	-3.11541000	-2.90180400	-0.10058900
O	4.13536400	-1.55223500	0.16175200
N	1.63645600	-0.86986100	1.66617300
O	0.53494000	-0.42969200	2.02299700
O	2.28510200	-1.74125600	2.22993700
N	-1.02642800	-1.41480900	-1.64597600
O	-0.12999800	-0.63898000	-2.00498500
O	-1.34197000	-2.46088100	-2.19740000

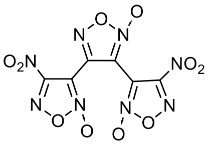

BNTFO-IV

O	-2.25658300	-2.15348600	1.44424500
O	-0.10303200	2.91968700	-0.14182100
O	-2.49668600	-2.19153900	-1.18931600
O	2.07506000	2.63871700	0.54471600
N	1.10737300	-1.26211000	1.28924000
N	3.29515200	-1.64880000	0.73968000
N	1.03266300	2.09926300	0.23363000
N	-1.15166500	2.10771400	-0.42089900
N	-1.29669600	-1.36345900	-1.07108100
N	-3.54326700	-1.51022100	-0.66690800
C	2.86695600	-0.55475000	0.17939400
C	1.50856100	-0.26433100	0.48875200
C	0.62286300	0.82424800	0.14718500
C	-0.75030300	0.86988800	-0.24629600
C	-1.67714100	-0.22855500	-0.47338800
C	-3.07428700	-0.37104500	-0.24888300
O	-0.24093900	-1.79846800	-1.49304800
O	0.06014300	-1.54988600	1.83810100
N	3.73373700	0.17624000	-0.74737600
O	3.14553900	0.90205900	-1.54667700
O	4.93625600	-0.01219600	-0.66434000
N	-3.96295800	-0.01219600	-0.66434000
O	-3.43936300	1.18286800	1.37832300
O	-5.12859500	0.59723300	0.10223700

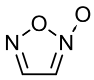

N	-0.76129000	0.09509300	-0.00013400
N	1.38213500	-0.71964000	-0.00004400
O	-1.97612900	-0.03902100	0.00007300
O	0.07236400	-1.10666000	0.00003300
C	0.08240800	1.12243100	0.00004000
C	1.39182200	0.59012600	0.00002100
H	2.32894700	1.12875000	0.00025600
H	-0.29012300	2.13318000	-0.00021200

CH_3NO_2

C	1.32811200	-0.00011800	-0.00305400
H	1.66769200	0.90639600	-0.49998300
H	1.66758700	-0.90765300	-0.49817000
H	1.63493100	0.00097200	1.04498300

N	-0.17115700	0.00000000	-0.01028200
O	-0.73391800	-1.09209400	0.00271700
O	-0.73368000	1.09221700	0.00271700

CH_3CH_3

C	0.00000000	0.00000000	0.76604800
H	0.50997400	0.88394300	1.16513600
H	0.51053000	-0.88362200	1.16513600
H	-1.02050400	-0.00032100	1.16513600

C	0.00000000	0.00000000	-0.76604800
H	1.02050400	-0.00032100	-1.16513600
H	-0.51053000	-0.88362200	-1.16513600
H	-0.50997400	0.88394300	-1.16513600

CH_4

C	0.00000000	0.00000000	0.00000000
H	0.63088200	0.63088200	0.63088200
H	-0.63088200	-0.63088200	0.63088200
H	-0.63088200	0.63088200	-0.63088200
H	0.63088200	-0.63088200	-0.63088200

References

1. Frisch, M. J. *et al*. Gaussian 09, rev. A.02, Gaussian, Inc., Wallingford, CT (2009).
2. Dennington, T. K. R & Millam, J. GaussView 5, V5.0.8, Semichem Inc., Shawnee Mission (2009).
3. Parr, R. G. & Yang, W. Density Functional Theory of Atoms and Molecules, Oxford University Press, New York (1989**).**
4. Head-Gordon, M., Pople, J. A. & Frisch, M. J. MP2 energy evaluation by direct methods. *Chem. Phys. Lett.* **153**, 503-506 (1988).
5. Linstrom, P. J. & Mallard, W. G. Eds. NIST Chemistry WebBook, NIST Standard Reference Database, 69, National Institute of Standards and Technology (2005).
6. Ochterski, J. M., Petersson, G. A. & Montgomery, J. A. A complete basis set model chemistry. V. Extensions to six or more heavy atoms. *J. Chem*. *Phys*. **104**, 2598−2619 (1996**).**
7. Montgomery, J. A., Frisch, M. J., Ochterski, J. M. &Petersson, G. A. A complete basis set model chemistry. VII. Use of the minimum population localization method. *J. Chem*. *Phys*. **112**, 6532−6542 (2000).
8. Westwell, M. S., Searle, M. S., Wales, D. J. & Wiliams, D. H. Empirical Correlations between Thermodynamic Properties and Intermolecular Forces. *J. Am. Chem. Soc.,* **117**, 5013−5015 (1995).
